# Medication related osteonecrosis of the jaws (MRONJ): Factors related to recurrence after treatment with surgery and platelet rich plasma (PRP) placement

**DOI:** 10.4317/medoral.24007

**Published:** 2021-10-27

**Authors:** Celia Sánchez-Gallego Albertos, José Luis Del Castillo Pardo de Vera, Aurora Viejo Llorente, José Luis Cebrián Carretero

**Affiliations:** 1 Department of Maxillofacial Surgery, La Paz Hospital, Madrid, Spain; 2 Head of section of Haematology and Hemotherapy, La Paz Hospital, Madrid, Spain; 3 Head of department of Maxillofacial Surgery, La Paz Hospital, Madrid, Spain

## Abstract

**Background:**

Medication-related osteonecrosis of the jaws (MRONJ) is a well-known complication associated with antiresorptive and antiangiogenic therapies. The purpose of this study was to analyse if there is any predictive factor of recurrence after local debridement plus platelet rich plasma (PRP) placement in MRONJ patients.

**Material and Methods:**

Seventy MRONJ patients treated at the department of Oral and Maxillofacial Surgery in La Paz Hospital (Madrid, Spain) were included in this retrospective study. All of them were treated surgically by local debridement and PRP placement. The observation period was between January 2012 and January 2019. Information regarding use, type, administration, and duration of therapy with BP/denosumab was recorded. The follow-up period ranged from 2-52 months. A descriptive analysis, a bivariate and a multivariate study were performed.

**Results:**

Most of the patients were women (82.9%) between 50-70 years old (64.3%), with a stage II disease (74.3%). The therapy lasted more than 12 months in 54.8% of them. Zoledronic acid was the main antiresorptive used (44.3%), followed by oral administered BPs (29 patients, 41.4%) and denosumab (10 patients, 14.3%). Osteoporosis (48.6%), breast cancer (30%) and multiple myeloma (11.4%) were the main diseases because the patients were taking antirresorptives. 13 patients (18.6%) experienced recurrence. We found that breast cancer patients (*p*>0.0001), smokers (*p*>0.016), and administration of zoledronic acid (*p*>0.0001) were related to recurrence. After performing the multivariate model, we found that the only factor related to recurrence was smoking habit (Wald 3.837, *p*=0.05, OR 6.12).

**Conclusions:**

recurrence after local debridement plus PRP placement in our MRONJ series affected to 18.6% of patients. It seems to be more frequent in breast cancer patients, smokers, and after zoledronic acid administration. Smoking habit was the only independent factor related to recurrence in our series.

** Key words:**Osteonecrosis of the jaw, recurrence, risk factor, bisphosphonates, zoledronic acid, denosumab, platelet rich plasma.

## Introduction

MRONJ is a well-known complication associated with antiresorptive and antiangiogenic therapies (1-3). These drugs are often used to treat bone disorders caused mainly by bone metastases (in cases of breast, lung, and prostate cancers), malignant hypercalcemia, and osteoporosis. It was reported for the first time by Marx in 2003 and currently, is defined as an exposed bone or bone that can be probed through an intraoral or extraoral fistula in the maxillofacial region that has persisted for longer than 8 weeks in a patient with current or previous treatment with antiresorptive or antiangiogenic agents, and without history of radiation therapy to the jaws or metastatic disease to the jaws (1,4). The current American Association of Oral and Maxillofacial Surgeons (AAOMS) staging system assigns patients to different stages of the disease based mainly on clinical criteria, and establishes a stage-specific treatment protocols, to select the appropriate treatment strategy for each patient (4). In broad terms, managing MRONJ patients can be particularly challenging because many surgical and medical interventions may not eradicate this process, but it is important to adequately identify the stage of disease of the patient to perform the best treatment possible to eliminate clinical symptoms, because the quality of life diminishes as the stage of disease increases, and is most marked between stages one and two (5,6).

In 1999, Anitua proposed for the first time the use of PRP in dentistry (7). Many authors have proposed an approach based on surgical debridement combined with the use of PRP to accelerate and improve healing (8-16). Adding PRP to the surgical treatment in patients with MRONJ reduces the recurrence rate as compared with control, with satisfactory healing in 85-90% of patients (8-9). Surgical debridement and PRP placement have achieved satisfactory results in many series, with recurrence rates ranging from 0 to 9% (10-12). Several studies have focused on the risk factors for developing MRONJ, being the dose, duration, and type of antiresorptive drug administered the principal risk factors (17-18). Obesity and smoking were recently associated with MRONJ, but the same study showed that the most relevant risk factor was zoledronic acid use (19). Nevertheless, factors related to recurrence after surgical treatment have not been thoroughly studied. A recent study demonstrated that extensive surgical treatments were related to a lower rate of recurrence in MRONJ patients, although this study did not include patients treated with the combination of surgery plus PRP placement (20).

The purpose of our study was to analyse the clinical and demographic characteristics of 70 patients diagnosed of MRONJ and treated with local debridement combined with PRP placement, at the Department of Oral and Maxillofacial Surgery in La Paz Hospital (Madrid, Spain). We wanted to determine the rate of recurrence in this group of patients and analyse if there is any risk factor for recurrence.

## Material and Methods

Seventy patients affected by MRONJ were included in this retrospective study. The inclusion criteria were: 1) Confirmed diagnosis of MRONJ based on clinical criteria (AAOMS 2014) and confirmed radiologically with OPG and CT scan; 2) management of the pathology with surgery and PRP placement; 3) availability of data regarding information about type of antiresorptive used, way of administration and duration of treatment; 4) surgery performed between January 2012 and January 2019.

Information regarding sex, age, comorbidities (hypertension, diabetes mellitus, steroid intake, smoking habit), pathology (disease why the patient was taking BP/denosumab), trigger of the ONJ, symptoms at diagnosis, stage of the disease at diagnosis, type of drug administered and duration of therapy, site of ONJ, presence of actinomyces in the bone extracted during surgery, and presence or absence of recurrence, defined as development of symptoms of ON and bone exposure confirmed radiologically. Patients were classified in 3 groups of age (<50, 50-70, >70 years old) to ease the statistical analysis. Staging of the ONJ was made according to the 2014 update of the American Association of Oral and Maxillofacial Surgeons’ position paper on MRONJ (4).

Surgery consisted in resecting all the necrotic bone, sequestrae and refreshing bone margins with a drill. In cases where teeth were present, the teeth were extracted if they were near the necrotic bone. The resection margins were determined intraoperatively, and drilling was stopped when bleeding bone was seen. The bone surfaces were hen covered with the PRP gel, and hermetic closure of the bone surface with mucosal flaps was always tried. In all cases, simple detached stitching was performed with resorbable material. In patients presenting with pathologic fracture, reduction plus osteosynthesis using a reconstruction plate with an extraoral approach was performed.

- Statistical analysis

The statistical analysis was structured in three phases:

A first one where a descriptive approximation of the study population was made, in all the variables that had been collected.

In the second part we proceeded to carry out a bivariate study to analyse the relationship of each characteristic collected with the dependent variable (recurrence). For that purpose, we used the Chi-square Test. Likewise, we used the binary logistic regression model, with the aim of quantifying the effect of each independent factor on the recurrence through the Odds Ratio (OR). In all these inferential statistical tests, significance is considered when *p*<.05.

Lastly, we performed a multivariate model with the factors that exert significative effect on the recurrence, obtained in the univariate model.

## Results

Demographic results are showed in Table 1. The sample comprised 70 patients (58 women (82.9%), and 12 men (17.1%)). Most of the patients were between 50-70 years (45 patients, 64.3%).

**Table 1:**
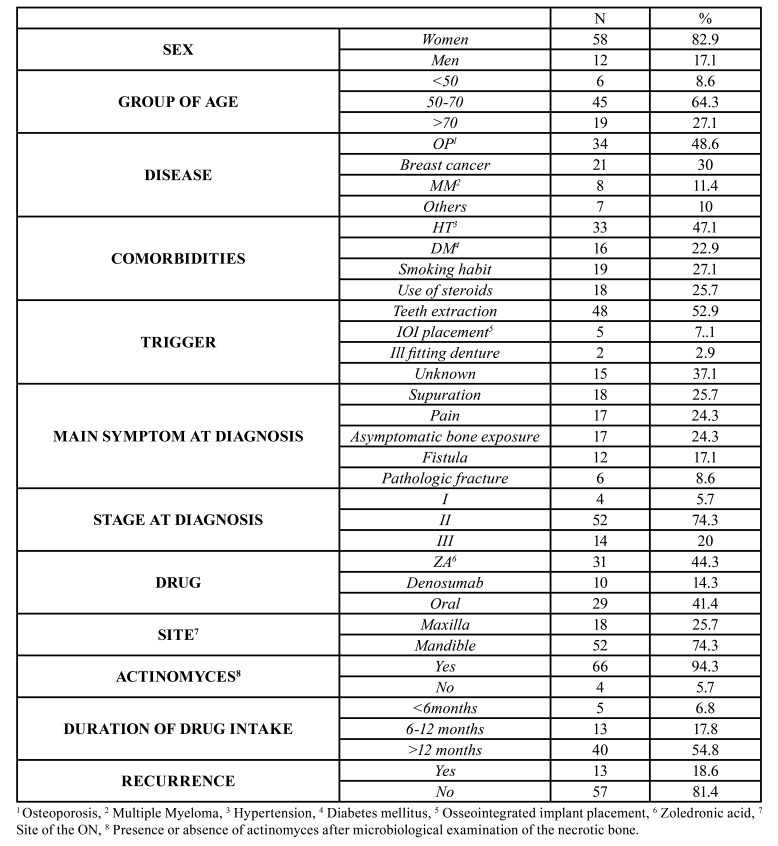
Descriptive analysis. Clinical characteristics of the patients included in our study, with diagnosed MRONJ.

Most of the patients had a stage II disease (74.3%). 54.8% of the patients had undergone treatment with antiresorptive for periods of more than 12 months. The main diseases for the administration of BPs were osteoporosis (34 patients, 48.6%), breast cancer (21 patients, 30%), and multiple myeloma (8 patients, 11.4%). The main antiresorptive used in our patients was zoledronic acid (31 patients, 44.3%) followed by oral administered BPs (29 patients, 41.4%) and denosumab (10 patients, 14.3%). 13 patients (18.6%) experienced recurrence.

After performing the Chi-Square test, we found that breast cancer patients (*p*<0.0001), smokers (*p*<0.016), and patients that had been administered zoledronic acid (*p*<0.0001) significatively experienced more recurrence (Table 2).

We included these factors in the multivariate model. The results are shown in Table 3. We found that the only factor that exert a significative effect on recurrence was smoking habit (Wald 3.837, *p*=0.05, OR 6.12).

**Table 2:**
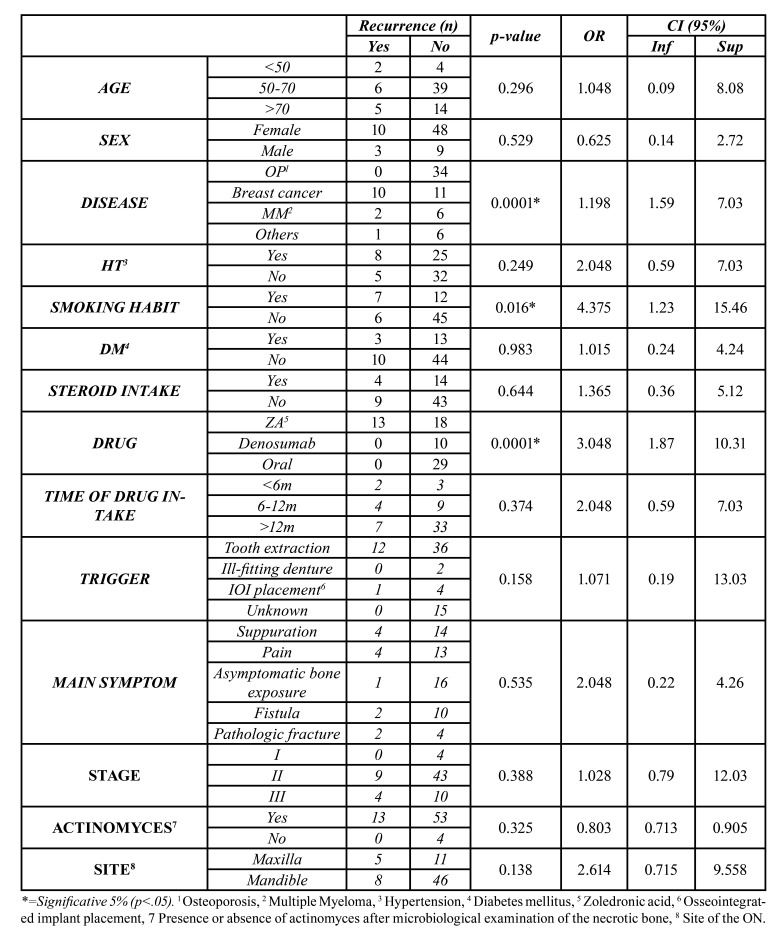
Bivariate analysis. Univariate effect of every characteristic about Recurrence - Yes/Recurrence-No. Chi Square test and logistic regression.

**Table 3:**
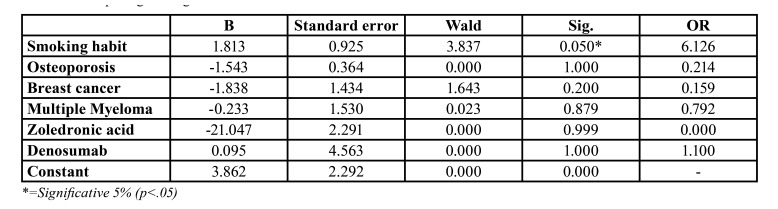
Multiple logistic regression model. Predictive factors of recurrence in cases of MROJN.

## Discussion

This study retrospectively assessed factors that predispose to a recurrence after surgical treatment with local debridement and PRP placement in a group of patients treated in the department of Oral and Maxillofacial Surgery in La Paz Hospital (Madrid, Spain). Several clinical studies have identified potential predisposing risk factors for developing MRONJ, such as the use of intravenous instead of oral BPs, treatment with glucocorticoids, the presence of comorbidities such as obesity, alcohol, or tobacco abuse, poor oral hygiene and periodontitis, and length of exposure to BP treatment (18,19,21-23). Factors related to recurrence after surgical treatment have not been thoroughly studied.

Our findings demonstrate that smoking habit, previous zoledronic acid administration, and breast cancer disease tend to produce a higher risk of recurrence after surgical treatment plus PRP placement in MRONJ patients. Smoking habit has been linked with numerous systemic pathologies and conditions. In the oral cavity, tobacco abuse delays wound healing, worsens periodontal disease, and promotes formation of premalignant lesions. Nevertheless, its role in ONJ has not been well established yet. In a retrospective study smoking habit demonstrated to negatively influence MRONJ staging (OR 1.80, 95% CI 1.03-2.80; *p*= 0.04) (18). In a case-control study, tobacco use approached statistical significance as a risk factor for ONJ in patients with cancer (OR = 3.0; 95% CI, 0.8-10.4) (19). On the contrary, in a more recent case-controlled study, tobacco use was not associated with ONJ in a sample of patients with cancer exposed to zoledronate (20). In our analysis, tobacco abuse was the only factor related to recurrence after performing the multivariate analysis. It has been proved that aggressive oral hygiene reduces the rate of ONJ in cancer patients. In a case series of 1243 cancer patients receiving BP or denosumab, the incidence of MRONJ was reduced from 4.6% to 0.8% by the implementation of regular dental check-ups and improved oral hygiene (24). As tobacco abuse worsens periodontal disease and oral health, it is not surprising that patients with a history of tobacco abuse have an increased risk of recurrence.

Incidence of MRONJ seems to be related to the dose, duration, and type of antiresorptive drug administered, being the long exposure to intravenous BPs the greatest and most consistent risk factor for the development of MRONJ (18). According to AAOMS studies, the frequency of MRONJ in oncology patients receiving high doses of BPs or denosumab is estimated at 1%-15%, and the frequency in the osteoporosis patient population receiving lower doses of BP or denosumab is much lower, estimated at 0.001%-0.01% (17). Our analysis shows that patients who underwent therapy with zoledronic acid, an intravenous administered BP, presented a higher risk of recurrence after surgery, even though this association did not show statistical significance in the multivariate model. Some authors demonstrated that zoledronic acid delays wound healing of the tooth extraction socket, producing an inhibition of new bone formation and reduction of the vascularization, suggesting that this BP inhibits the angiogenesis critical to the healing of the tooth extraction socket (23). On comparing denosumab with BPs in cancer patients, the associated ONJ rate is like that observed with BPs (25,26). Nevertheless, a recent study found that denosumab is associated with a significantly higher risk of developing MRONJ compared to zoledronic acid (27). We think that the role of type if antiresorptive drug administered in terms of recurrence after surgery deserves more investigation.

Breast cancer is the most frequently observed type of invasive cancer, affecting approximately 1 million women worldwide. BPs are some of the most effective treatments for preventing skeletal related events in this group of patients, being zoledronic acid the most effective one. In our series, patients with breast cancer presented more recurrence than patients with other pathologies, finding that was only proved in the univariate model (*p*<0.0001). A study showed that the risk of developing MRONJ in breast cancer patients could be major with the concurrent administration of trastuzumab, which is one of the most used agents for the management of metastatic breast cancers (28). The concurrent administration of trastuzumab was no investigated in our study, but it could have contributed to the increased rate of recurrence in the breast cancer patients. It is also possible that the fact that most of our patients with breast cancer had been receiving zoledronic acid acted as a confounding factor and for that reason, no significance was achieved in the multivariate model.

Our study has some limitations, as the limited study sample and the lack of information regarding dosage of antiresorptive used. However, we believe that our findings can serve as a basis for future studies to determine the subgroup of MRONJ patients with the highest risk of recurrence after surgical intervention and thus be able to establish a closer follow-up plan for them.

## Conclusions

Surgical treatment consisting of local debridement and application of PRP may be helpful in the treatment of patients with a stage II-III disease, with a rate of 18.6% of recurrence in our series. It seems to be more frequent in breast cancer patients, smokers, and after zoledronic acid administration. Tobacco abuse seems to be the only risk factor associated with an increased risk of recurrence after local debridement plus PRP placement in patients with MRONJ stage II-III.
